# Fecal Microbiota Transplantation Is Associated With Reduced Morbidity and Mortality in Porcine Circovirus Associated Disease

**DOI:** 10.3389/fmicb.2018.01631

**Published:** 2018-07-23

**Authors:** Megan C. Niederwerder, Laura A. Constance, Raymond R. R. Rowland, Waseem Abbas, Samodha C. Fernando, Megan L. Potter, Maureen A. Sheahan, Thomas E. Burkey, Richard A. Hesse, Ada G. Cino-Ozuna

**Affiliations:** ^1^Department of Diagnostic Medicine/Pathobiology, College of Veterinary Medicine, Kansas State University, Manhattan, KS, United States; ^2^Department of Animal Science, University of Nebraska–Lincoln, Lincoln, NE, United States; ^3^Abilene Animal Hospital, PA, Abilene, KS, United States; ^4^Kansas State Veterinary Diagnostic Laboratory, Kansas State University, Manhattan, KS, United States

**Keywords:** microbiome, swine, porcine reproductive and respiratory syndrome virus, porcine circovirus type 2, fecal microbiota transplantation, porcine circovirus associated disease

## Abstract

Porcine circovirus associated disease (PCVAD) is a term used to describe the multi-factorial disease syndromes caused by porcine circovirus type 2 (PCV-2), which can be reproduced in an experimental setting through the co-infection of pigs with PCV-2 and porcine reproductive and respiratory syndrome virus (PRRSV). The resulting PCVAD-affected pigs represent a subpopulation within the co-infected group. In co-infection studies, the presence of increased microbiome diversity is linked to a reduction in clinical signs. In this study, fecal microbiota transplantation (FMT) was investigated as a means to prevent PCVAD in pigs co-infected with PRRSV and PCV-2d. The sources of the FMT material were high-parity sows with a documented history of high health status and robust litter characteristics. The analysis of the donated FMT material showed the absence of common pathogens along with the presence of diverse microbial phyla and families. One group of pigs (*n* = 10) was administered the FMT while a control group (*n* = 10) was administered a sterile mock-transplant. Over the 42-day post-infection period, the FMT group showed fewer PCVAD-affected pigs, as evidenced by a significant reduction in morbidity and mortality in transplanted pigs, along with increased antibody levels. Overall, this study provides evidence that FMT decreases the severity of clinical signs following co-infection with PRRSV and PCV-2 by reducing the prevalence of PCVAD.

## Introduction

Fecal microbiota transplantation (FMT) is the process by which fecal microorganisms are donated by a healthy individual and subsequently transplanted into a diseased individual. The actual process of fecal transplantation has been described for centuries, being reported as early as the 4th century in China for the treatment of diarrhea in humans (Zhang et al., 2012) and in the 17th century in Italy as transfaunation for the treatment of diseases in ruminants (Borody et al., 2004). The recent surge in FMT usage and application is evidenced by a search in PubMed, where publication results for either “FMT” or “fecal microbiota transplant” comprise approximately 1,200 publications, with almost all being published within the last decade (National Center for Biotechnology Information [NCBI], 2017). For several human disease states, FMT has been shown to improve treatment outcome or resolve complex disease conditions. Although recurrent *Clostridium difficile* infections are by far the most frequent use for the application of FMT, other FMT-treatable digestive diseases include inflammatory bowel disease, ulcerative colitis, and metabolic syndrome (Borody and Campbell, 2011; Vrieze et al., 2012; Weingarden and Vaughn, 2017). The mechanism by which FMT is effective for the treatment of digestive diseases is likely associated with the restoration of normal flora. FMT is also associated with the improvement of non-digestive diseases, such as neurologic and respiratory disorders. For these conditions, the mechanism for the beneficial effect is likely more complex. For example, FMT restored the production of cytokines, including TNF-α and IL-10, in antibiotic-treated mice after respiratory infection with *Streptococcus pneumoniae* (Schuijt et al., 2016).

A different and less-explored role for FMT is in the prevention of disease. Traditionally, antibiotics and other growth promoters have been used in food animal production as prophylactics. Part of the benefit is likely derived from the maintenance of a microbiome that optimizes growth, either through individual species and/or metabolites, along with the prevention of common bacterial infections. As beneficial microbial populations are further characterized for their role in both growth and immunity, FMT or other microbiome therapeutics may provide an alternative to antibiotics and growth promoters in food animal production.

Porcine reproductive and respiratory syndrome virus (PRRSV) is the most costly disease of swine worldwide, with estimated annual losses to the US industry at $664 million, primarily due to respiratory disease and reduced weight gain in growing pigs (Holtkamp et al., 2013). Porcine circovirus type 2 (PCV-2) is also a significant and widely distributed pathogen of swine. PCV-2 is a causative factor associated with a group of disease syndromes termed porcine circovirus associated disease or PCVAD, characterized by muscle wasting, respiratory disease, jaundice, or pallor, and reduced weight gain in growing pigs (Segales, 2012). Models for PCVAD include co-infection with PRRSV and PCV-2, both of which cause systemic infections primarily targeting pulmonary and lymphoid tissues (Trible et al., 2012; Niederwerder et al., 2015, 2016).

Previous work utilizing a PRRSV/PCV-2 co-infection model has shown a consistent association between increased fecal microbiota diversity and improved outcome in nursery pigs (Niederwerder et al., 2016; Ober et al., 2017). Specifically, both pre- and post-infection fecal microbiome diversity was associated with several improved outcomes after co-infection, including reduced clinical disease severity, reduced virus replication, decreased lung lesions, and improved weight gain. Increased microbiome diversity, as measured by a pan-microbial microarray, was correlated with a reduction in PCVAD clinical signs as well as improved growth in subclinically affected pigs.

The hypothesis tested in the current study was that prophylactic administration of FMT to 3-week-old weaned barrows prior to co-infection with PRRSV and PCV-2 would reduce clinical signs and pathology associated with PCVAD. The sources of the FMT material were high-parity sows with life-long histories of high-health status and efficient production characteristics, such as the absence of frequent antimicrobial treatment, low pre-weaning mortality rates, large healthy litters, and a lack of clinical disease. The results showed that FMT reduces the number of pigs affected by PCVAD, including a reduction in virus load and increased viral antigen-specific antibodies. Therefore, FMT provides a potential therapeutic for the prevention of disease.

## Materials and Methods

### Animals and Housing

The use of animals and viruses in research was performed in accordance with the Federation of Animal Science Societies (FASS) Guide for the Care and Use of Agricultural Animals in Research and Teaching, the USDA Animal Welfare Act and Animal Welfare Regulations, and approved by the Kansas State University Institutional Animal Care and Use Committees and Institutional Biosafety Committees. Ten pairs of barrow siblings (*n* = 20; 24 days of age upon arrival) were obtained at weaning from a single high health commercial source negative for PRRSV, *Mycoplasma hyopneumoniae*, and porcine epidemic diarrhea virus (PEDV). Sibling pairs were from 10 different sows and the piglets were not vaccinated for PCV-2. No prophylactic or therapeutic antibiotics were administered at weaning or within 1 week of arriving at Kansas State. All pigs were housed in two identical environmentally controlled rooms at the Kansas State University Large Animal Research Center and maintained under biosafety level 2 (BSL-2) conditions. Each sibling pair was divided into either the control or fecal microbiota transplantation (FMT) group; the two groups were balanced according to arrival weight. Pigs were housed in groups of 10 in a 9.1 square meter pen with raised slatted flooring. All pigs were given approximately 24 h to acclimate to their new environment prior to FMT or mock-transplant treatment. Pigs were given access to food and water *ad libitum*.

### Viruses

The PRRS virus (isolate KS62; GenBank accession no. KM035798) used to prepare the inoculum for this study originated from the lymph node of a pig with PCVAD due to co-infection with PRRSV and PCV-2 (Trible et al., 2012). PRRSV was isolated by propagation on MARC-145 cells and titrated as previously described (Niederwerder et al., 2015).

The PCV-2d virus was a field-derived isolate. Serum containing the field isolate was heat-treated to remove heat-labile pathogens and was subsequently used to infect cesarean-derived, colostrum-deprived (CD/CD) pigs. Lung, spleen, and liver samples were collected from the CD/CD pigs at 21 days post-infection (dpi) and tested by qPCR for PCV-2d. Quantification cycle (Cq) values of the tissues used to create the inoculum for the current study were 14.9, 14.2, and 14.2 for liver, lung, and spleen, respectively, from a single CD/CD pig. A 10% tissue homogenate was created from the described tissues in Eagle’s minimum essential medium (EMEM; Sigma-Aldrich) supplemented with 50 μg/ml gentamicin. Following centrifugation at 100 × *g* for 15 min at 4°C, the supernatant was heat-treated at 55°C for 45 min to inactivate heat-labile pathogens. Analysis of the supernatant using the Lawrence Livermore Microbial Detection Array (LLMDA) confirmed the inoculum was negative for other common pathogens, such as porcine parvovirus, PRRSV, swine influenza virus, and *Mycoplasma hyopneumoniae*, but remained positive for porcine endogenous retroviruses (data not shown), which are ubiquitous in swine. PCV-2d infectivity was titrated on swine testicle (ST) cells. Briefly, serial 10-fold dilutions of PCV-2d challenge stock were added in quadruplicate onto rapidly dividing ST cells in a 96-well tissue culture plate (BD Falcon). Dilutions were prepared in Eagle’s minimal essential medium (EMEM; Sigma-Aldrich) supplemented with 7% fetal bovine serum (FBS; Sigma-Aldrich) and 50 μg/ml gentamicin (Lonza). Following a 3-day incubation at 37°C in 5% CO_2_, cells were fixed and permeabilized with 80% acetone. Cells were then stained with a polyclonal anti-PCV-2b primary antibody and a fluorescein (FITC) AffiniPure Goat Anti-Swine IgG secondary antibody (Jackson ImmunoResearch Laboratories, Inc.). Infected cells were visualized using an inverted fluorescent microscope and the 50% tissue culture infective dose (TCID_50_/ml) was calculated using the method of Spearman and Karber (Finney, 1964).

To prepare the inocula for pigs, the stock viruses were mixed to yield a 2-ml dose consisting of 10^4^ TCID_50_ PCV-2d and 10^5^ TCID_50_ PRRSV in MEM. The 2-ml dose was split, with 1 ml being delivered intranasally and 1 ml being delivered intramuscularly.

### Fecal Microbiota Transplant

Two sows from a commercial farrow-to-wean farm in Kansas were selected as donors for the transplant material. This herd was negative for PRRSV and had recently undergone a *M. hyopneumoniae* elimination program. The two sows were selected based on several characteristics, including older age (average age 4.8 years), high parity (9 and 12 litters born prior to donation), large litters with a high percentage of born alive piglets (15.1 ± 2.0 total born; 95% born alive), low pre-weaning mortality, no history of fetal mummification, and no antibiotic treatment received within at least the last 15 months prior to donation. Pre-weaning mortality in these two sows was primarily attributed to crushing injuries. Lifetime number of weaned pigs was 101 and 131 for each sow, respectively. For this study, feces were collected during lactation and sows had not yet weaned their respective litters at the time. Feces were initially screened and confirmed as negative for gastrointestinal parasites using a fecal float qualitative exam by the Kansas State Veterinary Diagnostic Laboratory (KSVDL).

To prepare the FMT, fresh feces was collected naturally during defecation or manually from the rectum of the two sows. Feces were collected on a single time and day, mixed, and processed within approximately 3 h after collection, during which the fecal microbiota was concentrated and stored using a protocol adapted from the human FMT literature (Hamilton et al., 2012). Specifically, feces were weighed into 50 g aliquots and mixed in a standard commercial blender (Oster, Sunbeam Products Inc.) with 250 ml of sterile saline (0.9% sodium chloride irrigation USP, Braun Medical) until homogenized. The fecal slurry was then passed progressively through 2.0, 1.0, 0.5, and 0.25 mm stainless steel sieves into a sieve receiver (Fisherbrand^TM^). The filtered liquid was collected, aliquoted into 50 ml tubes, and centrifuged at 6,000 × *g* for 15 min. The supernatant was discarded and each bacterial pellet was resuspended in approximately 20 ml of sterile saline. All resuspensions were gently vortexed prior to mixing the concentrated microbiota in a large beaker. Glycerol (molecular biology reagent grade, MP Biomedicals^TM^) was added to create a 10% glycerol suspension and the transplant material was stored at -80°C until the day of transplantation. On the day of transplantation, the FMT material was thawed for 2 h on ice and kept cold prior to administration.

The FMT material was submitted to KSVDL for routine bacterial culture, including aerobic culture, anaerobic culture, and *Salmonella* enrichment. Species identification was attempted for all bacteria cultured. The FMT material was also fully characterized on the LLMDA.

### Experimental Design and Sample Collection

Approximately 24 h after arriving at Kansas State University, pigs were administered a fecal microbiota transplant or a mock transplant (CONTROL). Mock transplants were made of 10% glycerol in sterile saline. Transplants or mock-transplants were administered as 5 ml doses delivered once daily for seven consecutive days prior to co-infection. To administer the FMT or mock-transplant, 5 ml doses were delivered through flexible dispensing tips (6.4 mm Flexoject^TM^ Dispensing Tips, Innovet). Solutions were delivered slowly on the tongue or in the cheek pouch, allowing the pig to chew on the tip and naturally consume the material over 30 s to 1 min.

At 32 days of age, all 20 pigs were infected with PRRSV and PCV-2d. Body weights of individual pigs were collected upon arrival (-8) and on -7, 0, 7, 14, 21, 24, 28, 32, 35, and 42 dpi. Blood samples were collected from all pigs on -7, 0, 4, 7, 11, 14, 21, 28, 35, and 42 dpi. Fecal samples were collected from all 20 pigs on -7 and 0 dpi. In addition to these planned sample collection times, blood, feces, and weights were collected on the day of death or euthanasia. Pigs were humanely euthanized under the direction of the attending laboratory animal veterinarian if (1) pigs had greater than or equal to 20% weight loss, (2) pigs were moribund or nonresponsive to veterinary treatment, or (3) pigs had severe dyspnea or clinical disease that compromised animal welfare. At 42 dpi, all remaining pigs were humanely euthanized in accordance with the American Veterinary Medical Association Guidelines for the Euthanasia of Animals and complete necropsies were performed.

### Clinical and Pathologic Evaluation

All pigs were assessed daily for the presence of clinical signs associated with PRRSV/PCV-2 co-infection, such as dyspnea, tachypnea, ocular discharge or conjunctivitis, coughing or sneezing, nasal discharge, aural cyanosis, open mouth breathing, decreased body condition, muscle wasting, rough hair coat, lethargy, depression, joint effusion, lameness, diarrhea, and pallor or jaundice. Pigs were visually examined by a veterinarian or veterinary assistant on each day of the study period. Under the direction of the attending veterinarian, appropriate treatments were administered to pigs with moderate to severe clinical disease. Examples of clinical presentations where treatment was administered included (1) dyspnea and/or tachypnea, (2) mucoid rhinorrhea, (3) conjunctivitis with swelling, (4) pallor or jaundice with muscle wasting, and (5) lethargy or depression with pyrexia. Clinically affected pigs were prescribed parenteral antibiotics, such as ceftiofur hydrochloride or oxytetracycline. Any pig with overt clinical disease and a rectal temperature of ≥104°F was administered parenteral flunixin meglumine, a nonsteroidal anti-inflammatory drug. Other supportive care, such as oral or subcutaneous fluids, was administered for significantly dehydrated pigs under the direction of the attending veterinarian. Pigs with evidence of conjunctivitis were treated with triple antibiotic ophthalmic ointment (bacitracin–neomycin–polymyxin, Vetropolycin, Dechra). Clinical signs and treatments unrelated to PRRSV or PCVAD (e.g., lacerations, dermatitis, congenital hernias, etc.) were documented and monitored but were not included in the data analysis related to clinical outcome. Treatment days were numerated for individual pigs over time. Treatment days were counted as each day a pig was prescribed a parenteral therapeutic. Mortality rate was calculated from those pigs that died or were euthanized prior to the 42-day termination of the study. Morbidity rate was calculated as the number of pigs demonstrating clinical signs deemed by the attending veterinarian to require veterinary intervention and prescription of parenteral therapy.

At 42 dpi, all surviving pigs (*n* = 11) were humanely euthanized with intravenous pentobarbital sodium. A board certified veterinary pathologist, blinded to the source of the pigs, performed complete necropsies and histopathology. First, whole body weights were collected post-mortem. Second, lungs and trachea were removed *in toto* immediately after euthanasia and total lung weights were measured. The trachea was excised immediately distal to the larynx. Lung weight to body weight ratio was calculated as a measure of pulmonary pathology. Dorsal and ventral aspects of the whole lung were photographed (Canon EOS Rebel T6 DSLR) and digital images were evaluated after gross necropsy using a photo scoring system. Gross anatomical photo scores were reported as the percentage of whole lung affected by pneumonia (ranging from 0 to 100%). Scores were combined from five sections of the lung as previously described (Halbur et al., 1995). The photos were evaluated by a board certified veterinary pathologist who was blinded to the source of the lung pictures.

Tissues collected for histopathology included lung (one section from each lobe) and tracheobronchial lymph node. Additional tissues were collected at the pathologist’s discretion by evidence of gross lesions. Tissues were fixed in 10% neutral buffered formalin for at least 7 days, routinely processed in an automated tissue processor, embedded in paraffin, and stained with hematoxylin and eosin (H&E stain). Microscopic lung lesions were scored using a 0–4 system as previously described (Niederwerder et al., 2015, 2016). Degree of lymphoid depletion was scored using a 0–3 system as previously described (Niederwerder et al., 2016).

### PCV-2 Immunohistochemical Staining

Porcine circovirus type 2 antigen staining in paraffin-embedded tissue thin sections was performed by personnel in the KSVDL. Briefly, deparaffinized slide-mounted thin sections were first treated with proteinase K (1.2 mg/ml diluted in Bond Enzyme Diluent with 0.35% ProClin 950) for 10 min at room temperature (Bond Enzyme Pretreatment Kit, Leica Biosystems). Rabbit anti-PCV-2 antibody (Iowa State University) was diluted at 1:500 in PowerVision IHC/ISH Super Blocking (Leica Biosystems) and applied to the tissue section for 15 min at room temperature. Bound antibody was detected by incubation with 25 μg/ml Poly-AP anti-rabbit IgG (Leica Biosystems) in antibody diluent for 25 min at room temperature. The complex was visualized using Fast Red chromogen (Bond Polymer Refine Red Detection Kit, Leica Biosystems) and counterstained with hematoxylin.

### Measurement of PRRSV and PCV-2 Viremia

Viral DNA and RNA was extracted simultaneously from 50 μl of serum using Ambion’s MagMAX 96 Viral Isolation Kit (Applied Biosystems) in accordance with the manufacturer’s instructions. PRRS viral RNA was quantified using EZ-PRRSV MPX 4.0 Real Time RT-PCR Target-Specific Reagents (Tetracore) according to the manufacturer’s instructions. For consistency, each plate contained Tetracore Quantification Standards and Control Sets for use with EZ-PRRSV MPX 4.0 RT-PCR Reagents. All PCR reactions were carried out on a CFX96 Touch Real-Time PCR Detection System (Bio-Rad) in a 96-well format using the recommended cycling parameters. The PCR assay results were reported as log_10_ PRRSV RNA starting quantity (copy number) per 25 μl reaction volume.

Porcine circovirus type 2d DNA was quantified using SsoAdvanced Universal SYBR green supermix (Bio-Rad) as previously described (Niederwerder et al., 2015, 2016). The PCR assay results were reported as log_10_ PCV-2 DNA starting quantity (copy number) per 20 μl reaction volume.

### Microsphere Immunoassay for Detection of PRRSV and PCV-2 Antibodies

Porcine reproductive and respiratory syndrome virus nucleocapsid protein and PCV-2b capsid protein fragments (43-233 and 160-233) were cloned into the pHUE expression vector, as previously described (Trible et al., 2011). For protein expression, bacteria were grown in Luria-Bertani (LB) broth plus ampicillin (0.01 mg/ml) and incubated at 37°C with shaking. Once the OD^600^ reached 0.4–0.6, protein expression was induced by adding 1 ml of 0.1 M isopropyl β-D-1-thiogalactopyranoside (IPTG) to the culture and bacteria were harvested 4 h later. Bacteria were pelleted by centrifugation at 4,000 × *g* for 10 min. Soluble proteins were purified using the USB PrepEase Histidine-tagged Protein Purification Kit (Affymetrix) under non-denaturing conditions, according to the manufacturer’s directions. Purity was assessed by SDS–PAGE and total protein measured using the Bio-Rad Protein Assay.

Proteins were coupled to carboxylated Luminex MagPlex^®^ polystyrene microspheres according to the manufacturer’s directions. For the assay, approximately 2500 antigen-coated beads, suspended in 50 μl PBS with 0.05% Tween-20 and 4% goat serum (PBST-GS), were placed in each well of a 96-well polystyrene round bottom plate (Costar). Sera were diluted 1:400 in PBST-GS and 50 μl was added to each well. The plate was sealed and incubated for 30 min at room temperature with gentle shaking. After the incubation, the plate was placed on a magnet and beads were washed three times with 190 μl of PBST-GS. For the detection of IgG, 50 μl of biotin-SP-conjugated affinity purified goat anti-swine secondary antibody (IgG, Jackson ImmunoResearch) was diluted to 2 μg/ml in PBST-GS and 100 μl was added to each well. The plate was incubated at room temperature for 30 min and washed three times followed by the addition of 50 μl of streptavidin-conjugated phycoerythrin (2 μg/ml in PBST-GS; SAPE). After 30 min, the plate was washed and microspheres resuspended in 100 μl of PBST-GS. Microspheres were analyzed using a MAGPIX instrument (Luminex) and Luminex^®^ xPONENT 4.2 software. A minimum of 50 microspheres was used for the calculation of mean fluorescence intensity (MFI). The sample to positive (S/P) ratio was calculated as the MFI of sample minus MFI of negative control divided by MFI of standard positive control minus MFI of negative control.

### Microarray Analysis of FMT and Fecal Samples

The LLMDA was used to analyze microbiome composition and diversity of the transplant material and fecal samples. This array detects annotated sequences of microbes associated with infection of vertebrates within GenBank^®^, the National Institute of Health genetic sequence database. The version 7 of the LLMDA in the 4plex 180K probe format was used in this study. This version of the array targets 4,377 viruses, 5,457 bacteria, 327 archaea, 319 fungi, and 132 protozoa. The LLMDA oligonucleotide probes vary between 50 and 65 nucleotides in length and have roughly equivalent affinities for their complementary target DNA molecules (McLoughlin, 2011). Probes were designed to detect all sequenced microbial families with a large number of probes per sequence (average of 30 probes) to improve sensitivity in the evaluation of microbial nucleic acids in a variety of samples. The high-density oligo LLMDA microarray and statistical analysis method have been extensively tested in numerous studies for viral and bacterial detection in pure or complex environmental and clinical samples (Gardner et al., 2010; Rosenstierne et al., 2014; Jaing et al., 2015; Niederwerder et al., 2016; Ober et al., 2017).

The PowerViral^TM^ Environmental RNA/DNA Isolation Kit (MO BIO, San Diego, CA, United States) was used to extract DNA and RNA from the fecal samples. For each sample, approximately 250 mg of feces was added to 600 μl of PV1/β-mercaptoethanol in a glass beat tube included in the kit. Samples were homogenized and lysed by vortexing tubes for 10 min at maximum speed. Samples were further processed using the PowerViral^TM^ Kit protocol. All samples were eluted into 100 μl of RNase-free water. The purified nucleic acids were quantified using the Thermo Scientific^TM^ Nanodrop^TM^ spectrophotometer. For each sample, 10 μl of the extracted DNA and RNA was amplified using the random amplification procedure as previously described (Rosenstierne et al., 2014). The amplified cDNA and DNA was purified with the Qiaquick PCR purification columns (Qiagen) and quantified using the Nanodrop^TM^ spectrophotometer.

Approximately 1 μg of amplified cDNA and DNA were fluorescently labeled using a one-coloring labeling kit (Roche NimbleGen, Madison, WI, United States). Briefly, the samples were labeled using nick translation with Cy3-labeled random nonamer primers (TriLink Biotechnologies, San Diego, CA, United States) and Klenow DNA polymerase at 37°C for 2 h. The labeled DNA was precipitated in isopropanol, centrifuged for 10 min, and the pellet was washed and dried. The pellet was then reconstituted in 50 μl of RNase-free water and quantified using the Nanodrop spectrophotometer.

The Agilent Technologies Oligo aCGH/ChIP-on-Chip Hybridization kit (Santa Clara, CA, United States) was used to hybridize samples to the arrays. For each sample, 10 μg of fluorescently labeled DNA was mixed with blocking agent, hybridization buffer and nuclease-free water. The samples were then denatured at 95°C for 3 min, and incubated at 65°C for 3 min. Each sample was then immediately loaded onto the array and hybridized for approximately 40 h at 65°C in a microarray rotator oven (Agilent Technologies Inc., Santa Clara, CA, United States) set to a speed of 20. Microarrays were then washed using the standard manufacturer’s protocol with Oligo aCGH/ChIP-on-chip Wash Buffer 1 for 5 min at room temperature and Oligo aCGH/ChIP-on-chip Wash Buffer 2 for 1 min at 37°C (Agilent Technologies Inc., Santa Clara, CA, United States). Using the SureScan Microarray Scanner (Agilent Technologies Inc., Santa Clara, CA, United States), all arrays were scanned to a resolution of 3 μm.

Microarray data were generated from the microbe sequences using the CLiMax method developed at Lawrence Livermore National Laboratory (Gardner et al., 2010), at a detection threshold of ≥99%. The log likelihood for each of the positive targets is estimated from the BLAST similarity scores of the array feature and target sequences, together with the feature sequence complexity and other covariates derived from BLAST results.

Diversity of the fecal samples was measured by calculating the number of families and species detected in each sample. The mean number of families and species in the control and transplant groups as well as the affected and unaffected groups was compared prior to (-7 dpi) and after (0 dpi) FMT. Microbiome composition was compared between these groups at the level of phylum, family, and species.

### 16S rDNA Analysis of Fecal Samples

Pig fecal samples were collected in cryovials and stored at -80°C until shipment to the University of Nebraska–Lincoln for DNA extraction and bacterial community analysis. DNA was extracted using the manufacturer’s protocol for Mag-Bind^®^ Soil DNA 96 Kit (Omega Bio-tek, Inc.) with the following modifications: precipitation of nucleic acids was done by using sodium acetate, isopropanol, and ethyl alcohol. 0.1× volumes of 10 mM sodium acetate was added to each sample tube, which were vortexed and later incubated on ice for 5 min. Subsequently, 1 ml of ice-cold isopropanol was added and samples were incubated at -80°C overnight to precipitate the DNA. The following day, samples were centrifuged at 4°C for 15 min at 16,000 × *g*. The supernatants of the resulting samples were discarded and the nucleic acid pellet was washed with 0.5 ml of ice-cold 70% ethyl alcohol. The samples were centrifuged for 2 min at 13,000 × *g*, the residual supernatant was discarded, and the nucleic acid pellet was air dried for 3 min. The nucleic acid pellet was dissolved in a 0.45 ml of Tris (10 mM, pH 8) and incubated for 1 h at 4°C. For further purification of dissolved nucleic acids, the KingFisher (Thermo Fisher Scientific) robot was used with reagents from the Mag-Bind^®^ Soil DNA 96 Kit. The resulting DNA was used for the tag-sequencing of the V4 region of 16S rDNA using the universal bacterial primers described previously (Kozich et al., 2013). A 20 μl PCR reaction contained 1× Terra^TM^ PCR Direct Polymerase Mix, 0.5 μl Terra polymerase, 20 mM of each primer, and 20–50 μg of DNA. The cycling conditions for PCR were the same as previously described (Paz et al., 2016). The PCR product size was confirmed by agarose gel electrophoresis. Normalization of the amplified PCR products was performed with Just-a-Plate^TM^ 96 PCR Purification and Normalization kit (Charm Biotech, San Diego, CA, United States) according to the manufacturer’s protocol. Following normalization, 10 μl from each sample was pooled and concentrated using Nucleospin^®^ Gel and PCR Cleanup kit (MACHEREY-NAGEL Gmbh & Co. KG, Duren, Germany) and was eluted using 20 μl of elution buffer. This pooled and purified sample was analyzed in a Agilent 2100 bioanalyzer (Agilent Scientific Instruments, Santa Clara, CA, United States) using Agilent High Sensitivity DNA Kit (Agilent Technologies, Inc., Waldbronn, Germany) to ensure the quality and quantity of the targeted V4 region of 16S rDNA. The concentration of the DNA library was determined using the DeNovix QFX Fluorometer (DeNovix Inc., Wilmington, DE, United States) and using DeNovix dsDNA Fluorescence Quantification Assay (DeNovix Inc., Wilmington, DE, United States). The resulting 16S rDNA libraries were sequenced using the Illumina MiSeq platform utilizing the 2 × 250 paired end sequencing strategy using a MiSeq Reagent Kit V3 (Illumina Inc., San Diego, CA, United States).

Data processing was performed on a custom pipeline utilizing several publicly available software tools. The paired-end reads were assembled into contigs after quality filtering using MOTHUR v.1.38.1 (Schloss et al., 2009). Operational taxonomic units (OTUs) were generated from the quality filtered sequences using the UPARSE pipeline (USEARCH v7.0.1090) (Edgar, 2013) at a threshold of 97% identity. Chimeric sequences were removed using the ChimeraSlayer gold.fa as the reference database using UCHIME (Edgar et al., 2011). OTUs were aligned against the v128 (SILVA) database and mismatched sequences were discarded. A phylogenetic tree was generated using high-quality aligned sequences within MOTHUR v.1.38.1 using the Clearcut algorithm (Sheneman et al., 2006). Taxonomies to the identified OTUs were assigned using QIIME v.1.9.1 pipeline (Caporaso et al., 2010) with the Greengenes reference database (gg_13_5_otus). OTUs representing Archaea and Cyanobacteria were removed as Cyanobacterial reads may be a result of contamination of plant chloroplast (Giovannoni et al., 1988) and the archaea sequences may be biased as the primers used are not designed to universally amplify all archaea. Alpha diversity matrices (Chao1 and Observed OTUs) were calculated using the QIIME v.1.9.1 pipeline. The rarefaction of the OTU table was performed using QIIME v.1.9.1 (Caporaso et al., 2010) with the lowest number of reads. For the experiment, 27035 was used as the lowest depth. The difference in bacterial communities (beta-diversity) among transplanted and control pigs was determined using the QIIME v.1.9.1 pipeline using distance matrices (weighted UniFrac, unweighted UniFrac, and Bray Curtis) from the rarefied-OTU table.

### Statistical Analysis

For 16s rDNA sequencing, a three-way ANOVA (considering the effect of treatment, day, and animal) was performed on the Chao1 and observed OTUs to estimate bacterial richness among the transplanted and control pigs with open source statistical software R (R Core Team, 2013). The overall bacterial community differences among treatments were determined by applying the permutational multivariate analysis of variance PERMANOVA on the weighted UniFrac distance matrix. Principal coordinate analysis (PCoA) was used on all distance matrices to generate plots which displayed global treatment effects. The PERMANOVA analysis was performed using R (R Core Team, 2015) (adonis function vegan package) (Oksanen et al., 2015) in which treatment was considered as a fixed effect and animal (pig) as a random effect with the pig as the experimental unit. A core microbiome was determined for each treatment group by only selecting the OTUs present in 80% of the animals in each group (8/10). The core OTUs were used to identify differential OTUs between treatments using the linear discriminant analysis (LDA) effect size with LefSe (Segata et al., 2011). LefSe analysis was performed using default settings and differential OTUs from all pairwise comparisons to generate heatmaps in R (R Core Team, 2015).

All remaining statistical analyses were performed using GraphPad Prism 7.01 software (La Jolla, CA, United States). Mean viremia, antibody levels, and weight measurements were compared between groups using repeated measures analysis with multiple unpaired *t*-tests. Survival curves were compared using the Mantel–Cox test and daily morbidity rates were compared using the Fisher’s exact test. Microscopic lung and lymph node lesion scores were compared between groups using the Mann–Whitney *U*-test. Gross photo scores and lung weight to body weight ratios were compared using the unpaired *t*-test. Microbiome diversity and number of species within family were compared between groups using the Mann–Whitney *U*-test. Proportions of each group with individual species and families detected were compared using Fisher’s exact test.

## Results

### Characterization of Fecal Transplant Material

Several methods, including aerobic culture, anaerobic culture, microarray, and fecal float, were used to characterize the fecal microbiota transplant material. Fecal floatation for parasites confirmed feces were negative for parasites, including *Ascaris suum*, through standard diagnostic testing at KSVDL. Aerobic and anaerobic culture identified several culturable bacteria known to inhabit the gastrointestinal tract, including non-hemolytic *Escherichia coli*, *Bacillus altitudinis*, *Streptococcus alactolyticus*, *Enterococcus hirae*, non-hemolytic *Staphylococcus* sp., *Bacteroides vulgatus*, and *Clostridium perfringens*. Several additional anaerobic bacteria were cultured but were unable to be identified at the genus or species level; these bacteria included Gram negative coccobacilli, Gram positive long rods, and large Gram positive boxy rods. *Salmonella* enrichment culture was negative. The pan-microbial array detected the most diversity and absolute number of organisms, with 12 phyla, 33 microbial families, and 49 microbial species detected (**Table 1**). Microbes were very diverse and from the phyla Actinobacteria, Amoebozoa, Bacteroidetes, Basidiomycota, Euryarchaeota, Firmicutes, Fusobacteria, Proteobacteria, Spirochaetes, Synergistetes, and Tenericutes. Additionally, a single virus was detected in the family *Circoviridae*. The majority of species detected fell within the Proteobacteria phylum (16/49; 32.7%) with the second highest number of species falling with the Firmicutes phylum (9/49; 18.4%) and the third highest number of species falling within the Tenericutes phylum (6/49; 12.2%). Using the above methods, known swine pathogens were not detected.

**Table 1:** Microorganisms detected in the fecal microbiota transplant material by the pan-microbial detection array.^∗^

Phylum^‡^	Family	Genus species
Actinobacteria	*Bogoriellaceae*	*Georgenia* sp.
	*Nocardiaceae*	*Rhodococcus rhodnii*
Amoebozoa	*Entamoebidae*	*Entamoeba nuttalli*
Bacteroidetes	*Bacteroidaceae*	*Bacteroides graminisolvens*, *Bacteroidetes bacterium*
	*Cyclobacteriaceae*	*Algoriphagus marincola*
	*Prevotellaceae*	*Prevotella* sp.
	*Rikenellaceae*	*Rikenella microfusus*
Basidiomycota	*Ceratobasidiaceae*	*Rhizoctonia solani*
Euryarchaeota	*Methanobacteriaceae*	*Methanobrevibacter oralis*, *Methanobrevibacter smithii*
Firmicutes	*Carnobacteriaceae*	*Alkalibacterium* sp.
	*Clostridiaceae*	*Candidatus Clostridium anorexicamassiliense*, *Clostridiaceae bacterium*, *Clostridium* sp.
	*Clostridiales*	*Clostridiales bacterium*
	*Lachnospiraceae*	*Lachnospiraceae bacterium*
	*Lactobacillaceae*	*Lactobacillus amylovorus*
	*Peptostreptococcaceae*	*Clostridium bifermentans*, *Clostridium mangenotii*
Fusobacteria	*Fusobacteriaceae*	*Psychrilyobacter atlanticus*
Proteobacteria	*Anaplasmataceae*	*Candidatus Xenolissoclinum pacificiensis*
	*Bradyrhizobiaceae*	*Bosea* sp., *Bradyrhizobium* sp.
	*Campylobacteraceae*	*Campylobacter* sp., *Sulfurospirillum arcachonense*
	*Desulfovibrionaceae*	*Desulfovibrio alkalitolerans*
	*Helicobacteraceae*	*Helicobacter pametensis*
	*Legionellaceae*	*Legionella lansingensis*
	*Piscirickettsiaceae*	*Thiomicrospira kuenenii*, *Thiomicrospira* sp.
	*Pseudomonadaceae*	*Pseudomonas* sp., *Rhizobacter* sp.
	*Sphingomonadaceae*	*Sphingomonas* sp.
	*Vibrionaceae*	*Candidatus Photodesmus katoptron*
	*Xanthomonadaceae*	*Ignatzschineria larvae*, *Xanthomonadaceae bacterium*
Spirochaetes	*Spirochaetaceae*	*Borrelia parkeri*, *Spirochaeta* sp., *Treponema pedis*, *Treponema* sp.
Synergistetes	*Synergistaceae*	*Aminiphilus circumscriptus*
Tenericutes	*Acholeplasmataceae*	*Acholeplasma equifetale*, *Acholeplasma granularum*
	*Mycoplasmataceae*	*Mycoplasma conjunctivae*, *Mycoplasma fermentans*, *Mycoplasma iowae*
	*Spiroplasmataceae*	*Spiroplasma apis*
Virus	*Circoviridae*	Fur seal feces-associated circular DNA virus


*^∗^Only those microbes identified at the phylum, family, and genus level are included. ^‡^Organized alphabetically by phylum; order listed when family unidentifiable.*

### FMT Had No Effect on Pigs Prior to Co-infection

Upon arrival to Kansas State University, mean weight of the control group was 7.05 ± 1.46 kg and mean weight of the FMT group was 7.07 ± 1.39 kg (*p* = 0.99, unpaired *t*-test using repeated measures; **Table 2**). No significant difference in weight gain was noted during the transplantation or mock-transplantation time period, suggesting no detrimental effect of FMT on weight gain in unchallenged conditions; mean weights for control pigs and FMT pigs on 0 dpi were 7.6 ± 1.7 and 7.3 ± 1.4 kg, respectively (*p* = 0.85, unpaired *t*-test using repeated measures). FMT and control pigs appeared clinically within normal limits.

**Table 2:** Effect of FMT on weight gain prior to co-infection.^∗^

Weight on arrival (-8 dpi)		Weight after 7 days of FMT (0 dpi)	
	
Control	FMT	Control	FMT
4.73	5.41	5.09	5.32
5.05	5.59	5.23	5.77
5.82	5.82	5.86	6.05
6.77	5.91	7.50	6.23
7.27	6.14	7.91	6.64
7.36	8.23	8.23	8.27
7.64	8.23	8.41	8.45
8.27	8.32	8.59	8.45
8.73	8.45	9.59	8.68
8.91	8.64	9.64	8.95
Mean: 7.05	Mean: 7.07	Mean: 7.60	Mean: 7.28
*SD*: 1.46	*SD*: 1.39	*SD*: 1.67	*SD*: 1.40
*p* = 0.99		*p* = 0.85	


*^∗^Data are shown in kilograms. Statistics performed by unpaired *t*-tests using repeated measures analysis.*

## FMT Reduced the Number of PCVAD-Affected Pigs

**FIGURE 1:**
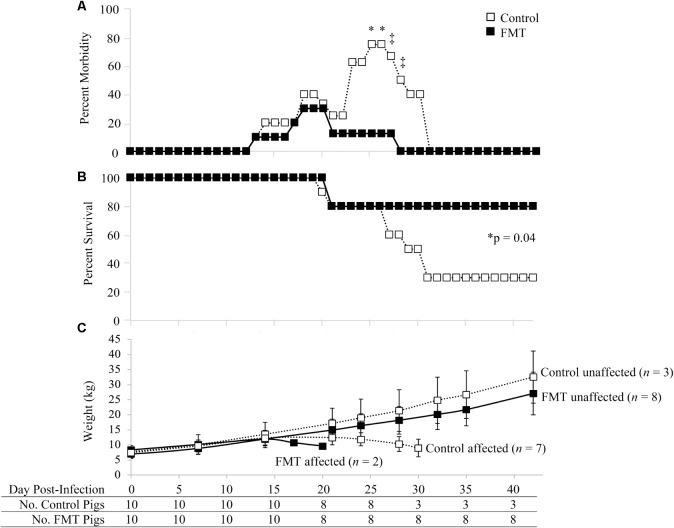
Morbidity and mortality of pigs with and without fecal microbiota transplantation prior to co-infection with PRRSV and PCV-2d. **(A)** Percent morbidity over time; data are shown as the percent of pigs in each group with veterinary treatment prescribed due to moderate to severe clinical disease. Asterisks demarcate statistically significant differences (^∗^p < 0.05 and ^‡^p < 0.1; Fisher’s exact test). **(B)** Survival curve shows a significantly higher survival rate in pigs administered the FMT. **(C)** Weight gain is shown as the mean ± standard deviation of control and FMT groups considered unaffected and affected by PCVAD, as identified by mortality and clinical disease. The surviving pigs in each group are shown at the bottom of the figure over time.

Morbidity and mortality of the FMT and control groups are shown in **Figure 1**. Morbidity rates of the control and FMT groups were comparable in the first 22 days after co-infection (**Figure 1A**). During this time, four control pigs and three FMT pigs showed clinical signs, including dyspnea, open-mouth breathing, coughing, tachypnea, mucoid rhinorrhea, conjunctivitis, reduced body condition, lethargy/weakness, and pyrexia. Starting on 23 dpi, the morbidity rates of the control and FMT groups diverged, with five control pigs (5/8; 62.5%) and only one FMT pig (1/8; 12.5%) exhibiting clinical signs sufficient to require veterinary intervention, including depression, dyspnea, tachypnea, coughing, open-mouth breathing, rough hair coat, mucoid oculonasal discharge, pyrexia, emesis and diarrhea, muscle wasting and loss of condition, ataxia, hypoxia, and cyanosis. On days 25 and 26 after co-infection, morbidity rates were significantly different (*p* = 0.04; Fisher’s exact test), with 75 and 12.5% of control and FMT pigs receiving treatment, respectively. A trend toward significantly higher morbidity was also seen on 27 dpi (*p* = 0.09; Fisher’s exact test) and 28 dpi (*p* = 0.05; Fisher’s exact test). By 28 dpi, clinical disease had completely resolved in the remaining eight FMT pigs while 50% of control pigs (3/6) remained on treatment. Clinical disease in affected FMT and control pigs (*n* = 9) was consistent with PCVAD. Unaffected pigs (*n* = 11) had mild to a complete lack of clinical signs. With regards to resulting mortality, initial death rates were similar between the two groups, with 20% mortality at 21 dpi. However, by the end of the study, the mortality in the control group was 70% compared to 20% for the FMT pigs (**Figure 1B**). Mortality between 19 and 30 dpi was due to pigs that died or were euthanized due to severity of clinical disease. Overall, the mortality rate of the control group (7/10, 70%) was significantly higher than that of the FMT group (2/10, 20%; *p* = 0.0447, Mantel–Cox test). When taken together, pigs which received the FMT treatment showed a reduction in PCVAD.

The clinically affected pigs showed a decrease in weight gain beginning at approximately 17 dpi (**Figure 1C**). Overall, no significant differences were detected between the two groups in regards to absolute weights during the biweekly or weekly weight measurements throughout the study (*p* > 0.05, repeated measures analysis with multiple *t*-tests). The one exception was the final weights on 42 dpi, where the three remaining control pigs had a mean weight significantly higher than that of the eight remaining FMT pigs (*p* = 0.04, repeated measures analysis). During peak clinical disease between 17 and 30 dpi, seven control pigs lost body weight whereas only two FMT pigs lost body weight (*p* = 0.07, Fisher’s exact test). Control pigs were 9.3 times more likely to lose weight during this period (95% CI: 1–56.5). Overall, FMT reduced the number of pigs which lost weight associated with clinical PCVAD.

**FIGURE 2:**
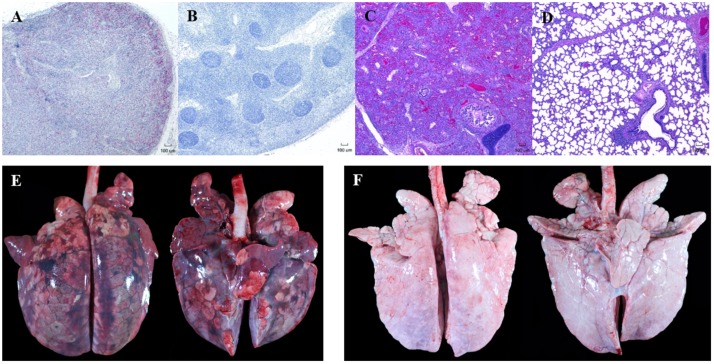
Representative gross and microscopic lesions associated with porcine circovirus associated disease (PCVAD). Images shown are from representative PCVAD-affected pigs with severe clinical disease between 19 and 30 dpi or minimally affected pigs for the purpose of comparison. **(A)** Immunohistochemical staining of a tracheobronchial lymph node showing severe lymphoid depletion associated with large amounts of PCV-2 antigen. **(B)** Immunohistochemical staining of a tracheobronchial lymph node showing lymphoid follicles with minimal lymphoid depletion and no PCV-2 antigen staining. **(C)** H&E-stained lung showing severe diffuse interstitial pneumonia affecting greater than 75% of lung. **(D)** H&E-stained lung showing mild and multifocal interstitial pneumonia affecting less than 50% of lung. **(E)** Dorsal and ventral gross lung showing severe consolidation, hemorrhage, and pneumonia affecting approximately 95% of lung. **(F)** Dorsal and ventral gross lung showing minimal consolidation, hemorrhage, and pneumonia affecting approximately 12% of lung.

Representative gross and microscopic lesions seen in pigs with PCVAD are shown in **Figure 2**. Images of minimally affected pigs are included for comparison. Examples of gross lesions included interstitial pneumonia with consolidation and hemorrhage, splenic infarcts, mucohemorrhagic rhinitis, lymphadenopathy with congestion and edema, pericardial effusion, mucohemorrhagic exudate in trachea, serous atrophy of fat, enteritis and intestinal ulceration, infarction of extremities, and tonsillar congestion. On gross examination of lung tissue, pneumonia was overall more severe in the control group, with 70% of the control pigs having severe interstitial pneumonia with marked edema, coupled with enlarged lymph nodes, characteristic of PCVAD. In addition, a few of the affected pigs in the control group had bronchopneumonia and fibrinous pleuritis. Interestingly, these pigs also had severe suppurative rhinitis. Rhinitis, bronchopneumonia, and pleuritis are suggestive of a secondary bacterial infection, likely due to immunosuppression associated with both PRRSV and PCV-2 infections. In contrast, only 30% of the FMT group had severe interstitial pneumonia and enlargement of the lymph nodes on gross examination, characteristic of PCVAD.

**FIGURE 3:**
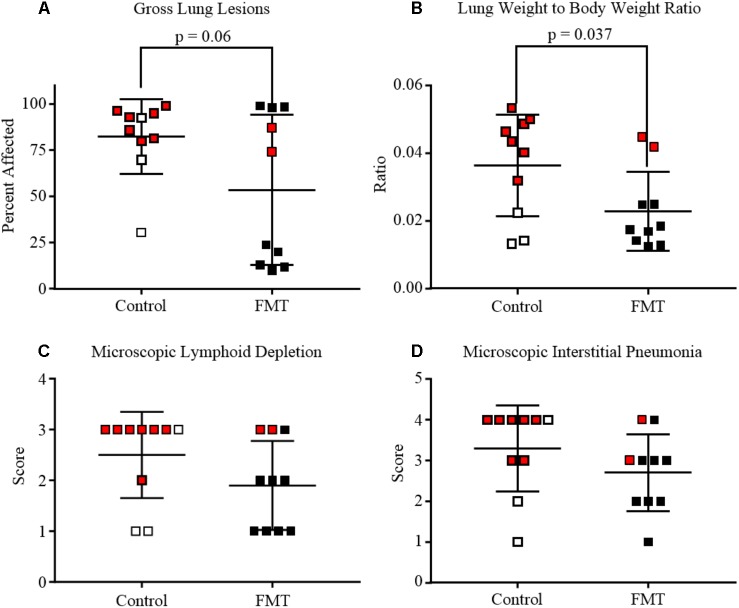
Degree of lung and lymphoid lesions in pigs after co-infection with PRRSV and PCV-2d. Data are shown as individual scores at the time of death with horizontal lines representing the mean ± 1 standard deviation for each group. Pigs shown in red are those that died or were humanely euthanized due to severity of disease. **(A)** Gross lung affected by pneumonia. Mean percent of lung affected was lower in FMT when compared to Controls (*p* = 0.06, unpaired *t*-test). **(B)** Lung weight-to-body weight ratio at the time of necropsy showing the control pigs had significantly higher ratios (*p* = 0.037, unpaired *t*-test). **(C)** Lymphoid depletion mean scores were higher in the control group (2.5 ± 0.3) when compared to the FMT group (1.9 ± 0.3), but differences were not statistically significant. **(D)** Microscopic lung lesion mean scores were higher in the control group (3.3 ± 0.3) when compared to the FMT group (2.7 ± 0.3), but differences were not statistically significant.

Gross lung tissue images were captured during necropsy and subsequently scored for severity of lesions (**Figures 2E,F**, **3A**). Control pigs had a range of 30.5–99.0% of lung affected, with a mean of 82.4 ± 20.3%. FMT pigs had a range of 10.0–99.0% of lung affected, with a mean of 53.6 ± 40.6%. These differences had a trend toward significance (*p* = 0.06, unpaired *t*-test). A lung weight-to-body weight ratio was calculated for each pig and is depicted in **Figure 3B**. Control pigs had significantly higher ratios when compared to FMT pigs (*p* = 0.04, unpaired *t*-test), indicative of increased cellular infiltrate and edema characteristic of interstitial pneumonia.

Lesions were also assessed through histopathology. Microscopic lesions in the lungs included lymphoplasmacytic and histiocytic interstitial pneumonia, suppurative bronchopneumonia, and interlobular septal edema and hemorrhage. Lymphoid depletion with histiocytic replacement was seen in the tracheobronchial lymph nodes. Lymphoid depletion was scored in all 20 pigs, with 7/10 control and 3/10 FMT pigs having severe lymphoid depletion (**Figures 2A**, **3C**). Although lymphoid depletion scores were generally higher in control pigs, this difference was not statistically significant (*p* = 0.155, Mann–Whitney *U*-test). Severe lymphoid depletion was associated with large amounts of PCV-2 antigen (**Figure 2A**). Degree of interstitial pneumonia was also scored in all 20 pigs, with 6/10 control and 2/10 FMT pigs having severe diffuse interstitial pneumonia with >75% lung lobe involvement (**Figures 2C**, **3D**). Control pigs again tended to have higher overall severity of microscopic lung lesions; however, this difference was not statistically significant (*p* = 0.164, Mann–Whitney *U*-test).

Taken together, the control pigs trended toward having more severe gross and microscopic lesions associated with PCVAD when compared to pigs that received the transplant material, indicating that FMT provided partial protection from both respiratory and lymphoid disease. This difference was seen due to an increased number of PCVAD-affected pigs in the control group.

### FMT Reduced PRRSV and PCV-2 Virus Replication and Increased Antibody Production

**FIGURE 4:**
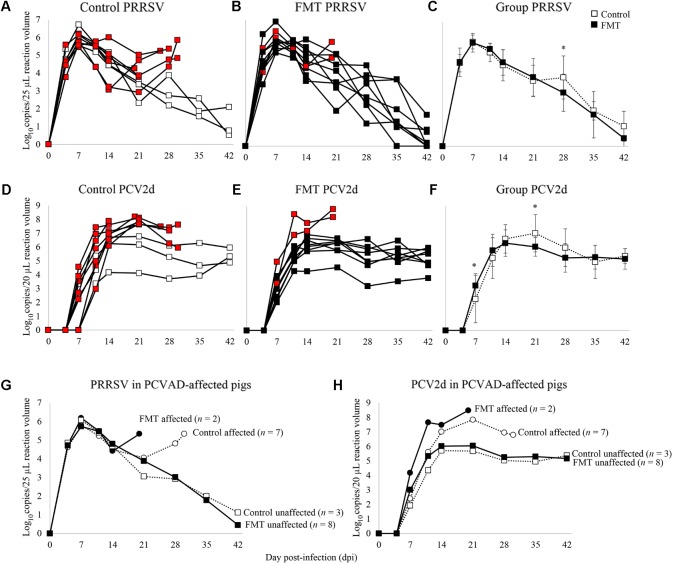
Time course of PRRSV and PCV-2d viremia. Data are shown as the log_10_ copy number/PCR reaction volume for individual pigs in both the control and FMT groups (**A,B** for PRRSV; **D,E** for PCV-2d). Red boxes indicate pigs that died or were humanely euthanized during the course of the co-infection trial due to severity of disease. **(**C,F**)** Data are shown as mean log_10_ copy number/PCR reaction volume ± 1 standard deviation for each group. Asterisks demarcate statistically significant differences for PRRSV and PCV-2d. **(**G,H**)** Data are shown as PRRSV and PCV-2 viremia in PCVAD-affected (circles) and PCVAD-unaffected (squares) pigs within the control and FMT groups, as measured by mortality and clinical disease.

Porcine reproductive and respiratory syndrome virus and PCV-2 viremia curves are shown for both individual pigs as well as group means (**Figure 4**). PRRSV viremia followed the typical time course, peaking at 7 dpi prior to a gradual decline over the next 5 weeks. Interestingly, most of the PCVAD-affected pigs had PRRS viremia rebound, a phenomenon initially described in 2010 (Reiner et al., 2010) and later by our group (Boddicker et al., 2012). For example, one control pig had peak PRRSV replication at 7 dpi (5.8 log_10_ copy number/25 μl reaction volume), a gradual decline of PRRSV replication until a low at 21 dpi (2.9 log_10_ copy number/25 μl reaction volume), and a second peak of PRRSV replication at 30 dpi (5.9 log_10_ copy number/25 μl reaction volume). When comparing mean PRRSV replication between groups, the only significant difference was seen at 28 dpi where the control group had significantly higher viremia; a mean of 3.9 and 3.0 log_10_ copy number/25 μl reaction volume was seen for control and FMT groups, respectively (*p* = 0.02, repeated measures analysis).

Porcine circovirus type 2 viremia also followed the typical time course, peaking later in the co-infection period between 14 and 21 dpi, followed by a plateau through the conclusion of the study. Interestingly, all nine pigs that died had significantly higher levels of PCV-2 replication when compared to pigs that survived the course of the study (**Figures 4D,E,H**). Specifically, surviving pigs maintained <7 log_10_ copy number/20 μl reaction volume in the serum at all measurements whereas pigs that died had >7 log_10_ copy number/20 μl reaction volume detected during the study. When comparing the two groups’ mean PCV-2 replication, significant differences were seen on 7 and 21 dpi, where the FMT group had a more rapid increase in PCV-2 replication on 7 dpi and a more rapid decline in PCV-2 replication on 21 dpi (*p* = 0.02 and 0.03, respectively; repeated measures analysis). Overall, PCVAD-affected pigs had high levels of PCV-2 and PRRSV in serum at the time of death or euthanasia, confirming the role of viral load in the course of clinical disease (**Figures 4G,H**). In general, virus replication during peak clinical disease was reduced in the FMT group, demonstrating a protective effect of FMT on viral load.

**FIGURE 5:**
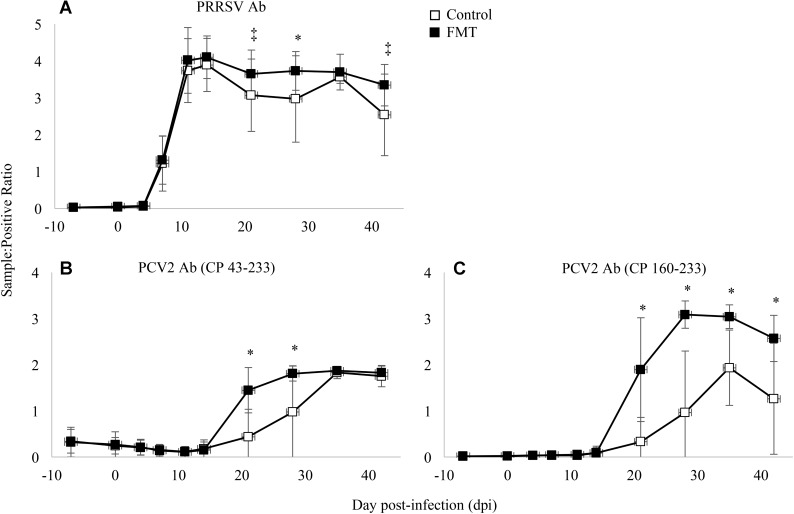
Detection of antibody in transplanted and control pigs. Data are shown as the mean sample:positive ratio ± 1 standard deviation for PRRSV **(A)**, PCV-2 large epitope **(B)**, and PCV-2 decoy epitope **(C)**. Differences between the two group are shown as ^∗^p < 0.03 and ^‡^p < 0.06 (repeated measures analysis using multiple *t*-tests).

Antibodies were measured against PRRSV N protein, PCV-2 whole capsid protein (CP 43-233), and PCV-2 decoy epitope (CP 160-233). PRRSV antibodies were detected similarly in both groups initially on 7 dpi and peaking between 11 and 14 dpi (**Figure 5A**). PRRSV antibodies were detected at a greater level in FMT pigs on 21, 28, and 42 dpi (*p* = 0.06, *p* = 0.02, and *p* = 0.05, respectively; repeated measures analysis). When comparing PCV-2 antibody levels (**Figures 5B,C**), a similar trend was noted with FMT pigs having higher antibody levels. FMT pigs had higher CP 43-233 antibodies at 21 and 28 dpi whereas the FMT pigs maintained higher CP 160-233 antibodies from 21 dpi until the conclusion of the study. Taken together, FMT promoted the production of higher and more sustained levels of antibodies directed at both PRRSV and PCV-2.

### FMT Shifted Microbiome Composition

Fecal microbiomes of the transplanted and control groups were analyzed both before and after FMT or mock-transplantation by a pan-microbial array (LLMDA) and 16S rDNA sequencing. First, microbiome composition and diversity were measured by the LLMDA. Diversity was calculated as the mean number of species and families in each group; after transplantation, the mean number of species was 62.3 ± 2.7 and 59.9 ± 4.2 for the control and FMT groups, respectively. With regards to family diversity, the mean number of families on 0 dpi in the control group was 35.3 ± 2.7 while the transplant group had 33.8 ± 2.6. Interestingly, no significant differences in species or family diversity were detected in the FMT group compared to the controls after transplantation using the pan-microbial array. Microbiome diversity, as measured by the microarray, was also similar in the two groups upon arrival (data not shown).

**FIGURE 6:**
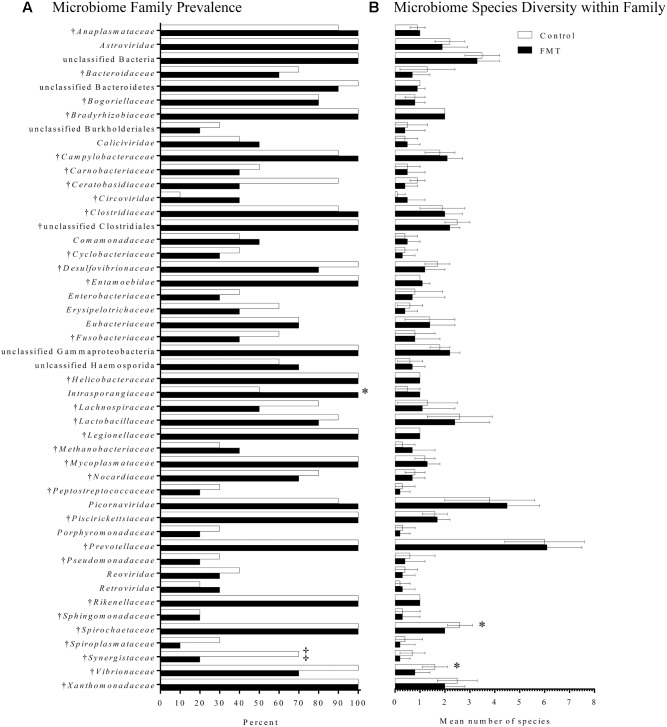
Fecal microbiome composition as detected by the pan-microbial array in FMT and control pigs after 7 days of transplantation. **(A)** Microbiome family composition is shown as the percent of FMT pigs (*n* = 10) and control pigs (*n* = 10) with each family detected on the pan-microbial array. Families with a total prevalence of <40% between the FMT and control groups are not shown. There was a significantly higher prevalence of a species within the family *Intrasporangiaceae* in the FMT group. A trend toward a higher percentage of control pigs having species within the family *Synergistaceae* was also detected (^‡^p = 0.07; Fisher’s exact test). **(B)** Data are shown as the mean number of species detected ± 1 standard deviation in each family identified in FMT and control pigs. Within the families *Spirochaetaceae* and *Vibrionaceae*, there was greater species diversity in the control group compared to the transplanted group. ^†^Indicates families found in the transplant material.

Microbiome composition was also assessed by the LLMDA through the presence of individual phyla, families, and species in the two groups. After 7 days of transplantation, there were 64 total families and 166 total species detected in both the control and transplanted groups. Several differences were detected between the transplant and control groups after transplantation that were not detected upon arrival (**Figure 6A**). Specifically, the family *Synergistaceae* was detected at a decreased prevalence rate in the transplanted group after FMT compared to the controls (20 and 70%, respectively; *p* = 0.07; Fisher’s exact test) and a bacterium in the *Intrasporangiaceae* family was detected in a higher proportion of the transplant pigs when compared to the control pigs (100 and 50%, respectively; *p* = 0.03; Fisher’s exact test). Even though members of the *Intrasporangiaceae* family have been discovered in environmental samples and sequenced (Weissbrodt et al., 2014; Zhao et al., 2017), there is a lack of research exploring the effects of these organisms on the vertebrate gut microbiome.

Finally, species diversity within each family was assessed for differences associated with transplantation (**Figure 6B**). Most families had similar species diversity between the control and transplanted groups. However, within the families *Spirochaetaceae* and *Vibrionaceae*, there was greater species diversity in the control group compared to the transplanted group. The mean number of species within the family *Spirochaetaceae* was 2.6 species in the control group, while in the transplant group it was 2.0 species (*p* = 0.01; Mann–Whitney *U*-test). The mean number of species within the family *Vibrionaceae* was 1.6 species in controls, while in the transplant group it was 0.8 species (*p* = 0.02; Mann–Whitney *U*-test). Overall, the LLMDA failed to detect a global increase in microbiome diversity after 7 days of transplantation; however, several shifts in microbiome composition were detected, primarily based on a reduction in bacteria generally considered pathogenic.

A secondary analysis was performed to assess microbiome diversity between PCVAD-affected and unaffected pigs using the LLMDA. Affected and unaffected pigs had similar mean numbers of families and species when compared on days -7 and 0 post-infection. However, there was a significant increase detected in the number of families in the unaffected pigs between -7 and 0 dpi, with the mean number of families detected increasing from 28.9 to 33.7 during the transplantation period (*p* = 0.03; Mann–Whitney *U*-test). Although an increase in family diversity was also seen in the affected pigs between -7 and 0 dpi, this difference was not statistically significant (*p* = 0.20; Mann–Whitney *U*-test). Species diversity also increased in both affected and unaffected pigs during the transplantation period, albeit at a similar rate (*p* = 0.07 and *p* = 0.09 for unaffected and affected pigs, respectively; Mann–Whitney *U*-test). Taking the results together from the LLMDA, there is some evidence suggesting that pigs unaffected by PCVAD after challenge had a greater increase in microbiome diversity during the transplantation period.

Fecal bacterial communities of the control and transplanted groups were also analyzed using 16S rDNA sequencing both prior to and immediately following transplantation (Supplementary Figures 1, 2). A total of 2,446,796 quality-filtered 16S rDNA sequences of the V4 region were generated with an average read depth of 61,169 reads per sample. To determine if sampling depth was adequate for gut microbiota analysis, Good’s coverages were calculated, which displayed that 99.4–99.8% of the bacterial community in the gut was represented in the dataset.

The Chao1 alpha-diversity metric for richness was similar in the transplant and control animals at 7 days post transplantation (*p* ≥ 0.82; Supplementary Figure 1A). The phylum level distribution of the major taxa in both control and transplanted pigs included Bacteroidetes, Firmicutes, and Proteobacteria. Interestingly, the phylum Actinobacteria was almost half in the transplanted group compared to the control group (1.7 vs. 3.3%, respectively; Supplementary Figure 1B). The family level analysis of the transplanted and control animals revealed *Prevotellaceae*, *Paraprevotellaceae*, Bacteroidales S24-7, *Lactobacillaceae*, *Christensenellaceae*, *Lachnospiraceae*, *Ruminococcaceae*, and *Veillonellaceae* to be the predominant families (Supplementary Figure 1C). Phyla composition was not significantly different across the transplant and control animals. Bacterial communities did not cluster by treatment group, suggesting no global shifts in the bacterial populations as a result of the FMT. However, there was a significant day effect displayed during transplantation (*p* < 0.001).

To reduce animal–animal variation, a core measurable microbiome (CMM) was defined for the control and transplanted groups. For the transplant group, the CMM was composed of 306 OTUs (23.5% of total OTUs), which represented 81.1% of the rarefied quality-filtered reads. For the control group, the CMM was composed of 316 OTUs (25.3% of total OTUs), which represented 83.2% of the rarefied quality-filtered reads.

To further investigate potential bacterial community differences across the transplanted and control groups, differentially abundant OTUs across the CMM were identified using the LefSe algorithm (Segata et al., 2011). A total of 30 significant, differentially abundant OTUs with LDA scores ≥2 were identified across comparisons of the two groups (Supplementary Figure 2). The differential OTUs associated with the transplant group belong to the bacterial families *Veillonellaceae*, *Lachnospiraceae*, and *Ruminococcaceae*.

Additionally, the 16S rDNA bacterial community composition was analyzed based on the prevalence of disease in the PCVAD-affected (*n* = 9) and unaffected (*n* = 11) pigs. A two-way ANOVA was performed on the Chao1 and observed OTUs to determine the effect of subsequent disease phenotype on pre-challenge bacterial diversity. Both measures of bacterial diversity were lower in the affected group on 0 dpi (Supplementary Figure 3); however, these differences were not statistically significant (*p* ≥ 0.14 and *p* ≥ 0.24, respectively). We also performed beta-diversity analysis to determine the effect of the disease phenotype on global microbial community diversity using PERMANOVA analysis. This analysis demonstrated no differences in community composition based on disease phenotype (*p* ≥ 0.46), but detected a significant effect of day (*p* < 0.001) on the bacterial community composition.

## Discussion

Although FMT has been accepted for centuries as a treatment for various gastrointestinal diseases, it has only been very recently that FMT has been recognized as an alternative therapeutic for diseases outside of the gastrointestinal tract, such as respiratory or neurologic diseases (Bakker and Nieuwdorp, 2017; Staley et al., 2017). Moreover, using FMT as a prophylactic tool prior to the development of disease has been even less explored. The current study describes FMT efficacy when used as a prophylactic tool to prevent PCVAD in pigs infected with two important swine pathogens. Additionally, the study was conducted in a manner in line with current swine industry standards, where pigs are typically handled at 3 weeks of age after weaning and without broad-spectrum antimicrobial therapy.

To identify beneficial characteristics of the FMT material, two diagnostic tests were used for characterization: (1) a pan-microbial array and (2) bacterial culture. Comparing these results reveals several discrepancies between the two different detection methods. Interestingly, several bacteria cultured through standard methods, such as *Escherichia coli* and *Streptococcus* sp., are not detected on the pan-microbial array. Culture methods may promote growth of certain well-characterized bacterial species, even if the genome is present at a rate lower than that detectable by the microarray. Previous studies have reported similar results. For example, Sung et al. (2018) reported bacterial species detected in bronchoalveolar lavage fluid by both conventional culture techniques as well as next-generation sequencing (NGS). Similar to the current study, they detected some species only by culture and other species only by sequencing, with increased diversity detected in the genome-based technique. Interestingly, the genera *Staphylococcus* and *Escherichia* were only detected by culture and not by NGS (Sung et al., 2018), a result similar to our findings on the transplant material. However, with these two species being common environmental microbes, contamination of the bacterial culture must also be considered. With advantages and limitations to each diagnostic test, using culture and DNA-based techniques in combination can serve to provide a more comprehensive characterization of complex microbial communities.

Biphasic clinical disease after co-infection with PRRSV and PCV-2 has been described previously (Niederwerder et al., 2015, 2016). Clinical disease associated with PRRS is typically seen in the first 21 dpi, during peak PRRSV replication. In contrast, clinical disease associated with PCVAD is typically seen after 21 dpi and is associated with the peak and plateau in PCV-2 replication. Although respiratory signs are common in both phases, clinical disease associated with PCVAD is typically more severe and associated with significant weight loss and muscle wasting. Compared to previous studies where PCV-2b was used, the current study used PCV-2d, which has been recently reported as the most common circulating PCV-2 genotype in US swine (Xiao et al., 2016). This use of PCV-2d appeared to increase morbidity and mortality rates of co-infected pigs. The principal effect of FMT in the co-infection model was to decrease the number of PCVAD-affected pigs, as demonstrated by a significant reduction in morbidity and mortality. Specifically, a 70% reduction in mortality of transplanted pigs was demonstrated. Additionally, parenteral antimicrobial treatments prescribed for clinical disease were reduced by 60% in the FMT group. With increasing pressure to eliminate antimicrobial usage in food animal production, a 60% decrease in prescribed antimicrobials is a significant effect, important to both human and animal health. Interestingly, FMT did not appear to significantly impact clinical disease in the first half of the co-infection period, typically associated with PRRS. Nevertheless, further research is warranted to understand if FMT improves response to PRRS in a PRRSV-only infection model.

The mechanisms by which FMT is effective are poorly understood but thought to be associated with increasing microbial diversity and restoring normal microbial communities which provide both local and systemic benefits to the host (Khoruts and Sadowsky, 2016). How FMT protected nursery pigs from developing PCVAD in this study is unknown, but may be due to several possible mechanisms. First, FMT increased the relative abundance of several bacterial families associated with metabolism, including *Veillonellaceae*, *Lachnospiraceae*, and *Ruminococcaceae*. The members of the *Lachnospiraceae* family are fermentative, anaerobic, chemoorganotrophic, and have the ability to hydrolyze many different substrates, such as xylanase, α- and β-glucosidase, and α- and β-galactosidase (Stackebrandt, 2014). Bacteria in the *Ruminococcaceae* family are common gut microbes of animals and humans, which help the host break down complex carbohydrates (Ben David et al., 2015). Both *Lachnospiraceae and Ruminococcaceae* have previously been associated with fatness traits in pigs (He et al., 2016) and our previous work detected a positive association between *Ruminococcaceae* species and growth after co-infection (Ober et al., 2017). In the current study, the comparison of absolute weights over time in the two groups was significantly impacted by the high mortality rate in the control pigs; as such, the increase in weight in the remaining control pigs at the conclusion of the trial was likely the result of decreased competition for feed. Evaluating the weight gain of individual pigs, it is clear that the weight gain of the control pigs was impacted to a greater extent than the FMT pigs throughout the trial.

As a second possible mechanism, FMT may modulate the systemic immune response, increasing the function of immune cells or stimulating cytokine production. In the current study, evidence for an enhanced immune response to both PRRSV and PCV-2 was demonstrated by a reduction in pulmonary pathology as well as a more robust and prolonged antibody response to both viruses detected in the serum of transplanted pigs. It is also possible that FMT may enhance gastrointestinal health and provide competitive exclusion of pathogens. Supporting this possible mechanism in the current study was the documented reduction of two bacterial families thought to be primary pathogens, including *Spirocheataceae* and *Vibrionaceae* (Tsinganou and Gebbers, 2010; Karami et al., 2014; Le Roux et al., 2015), in transplanted pigs.

Perhaps surprising in the current study was the lack of significant global increases of microbiome diversity in pigs receiving the transplant. In humans, where FMT is most commonly used to treat recurrent *C. difficile* infections, patients have almost always been treated with several standard doses of antibiotics, making an increase in microbiome diversity more likely with FMT therapy. Even in these human patients, however, Staley et al. (2016) reported that successful resolution of *C. difficile* infections through FMT treatment did not require complete microbiota engraftment (Staley et al., 2016). Similarly to the current study, it does not appear that complete microbiota engraftment occurred in transplanted pigs; nonetheless, significant beneficial effects occurred due to transplantation.

Compared to humans receiving FMT, an important concept to discuss for the current study is that pigs were not treated with antimicrobials and thus had normal microbiomes for their age at the time of transplantation. Pigs were weaned at 3 weeks and allowed normal contact with sows and a commercial environment after birth. The rationale behind this experimental design was to model commercial conditions, where antimicrobial stewardship practices have made it increasingly important to avoid the use of antimicrobials for prophylactic use, and to evaluate FMT as a preventative tool that may be applied to swine production in the field, including its use as a replacement for antimicrobials. However, this could be considered a limitation of the study, due to our inability to control the microbiota present at the time of transplantation, such as would be the case had microbiota-depleted or germ-free pigs been utilized.

A second consideration should be the lack of gender and donor diversity in the current study; the FMT material was collected from two older sows and transplanted into weaned barrows. Although the FMT donors were selected based on several specific characteristics and requirements, it should be noted that donation from other sows or boars may have resulted in a different outcome. Gender has been previously described as a factor affecting the success, failure, or effect of FMT. For example, Meighani et al. (2016) reported that female sex was a significant predictor of FMT failure when treating humans with recurrent *C. difficile* infection (Meighani et al., 2016). In contrast, Siegerstetter et al. (2018) demonstrated that female chickens trended toward having higher feed intake (*p* = 0.087) and weight gain (*p* = 0.081) after FMT; this same beneficial effect of FMT was not seen in male chickens (Siegerstetter et al., 2018). Regarding the current study, it is unknown how FMT collected from boars or administration to weaned gilts would have impacted the study outcome. Additional research is warranted to understand the effects of gender, both in collection and administration, on FMT efficacy in swine.

Decades of research into control of respiratory disease associated with PRRS have failed to produce a broadly protective vaccine or programs capable of long-term virus elimination from farms. Due to the significant economic and animal welfare impacts that respiratory disease continues to have on the swine industry, it is necessary to consider alternative strategies, such as FMT, for the control of respiratory disease in swine production. Very recently, microbiome therapeutics have been developed for the prevention and/or treatment of diseases in the respiratory tract of children. For example, in May 2017, a microbiome therapeutic utilizing a mixture of four gut bacteria, including *Faecalibacterium*, *Lachnospira*, *Veillonella*, and *Rothia* (FLVR), was announced for preventing childhood asthma and potentially other childhood allergic diseases (Arrieta et al., 2015; Anonymous, 2017). Interestingly, three of those bacterial families were differentially expressed in FMT pigs post-transplantation. Utilizing beneficial gut microbes for the prevention and treatment of respiratory disease is an emerging and exciting area of study. As respiratory infections are a major cause of morbidity and mortality in swine and other livestock, FMT or other microbiome therapeutics provide a promising approach for control of these complex, often polymicrobial, and economically devastating diseases.

## Conclusion

This study provides evidence of the significant relationship between the gut microbiome and outcome following systemic viral infections in swine. Most importantly, novel insight is provided into our ability to modulate the microbiome through FMT to improve the clinical outcome of pigs to common pathogens. Future research is necessary to understand the mechanism behind this relationship and how large-scale microbiome modulation could be adapted to increase the health of PRRS-positive herds in the field.

## Data Availability

The 16S rDNA sequence data have been deposited in the NCBI Sequence Read Archive under accession no.: PRJNA478067. Any additional raw data supporting the conclusions of this manuscript will be made available by the authors upon request to any qualified researcher.

## Author Contributions

MN conceived and designed the study, performed data analysis and interpretation, and wrote the manuscript. LC performed the microarray experiments, analyzed the data, and wrote the manuscript. RR designed the experiments, interpreted the data, and revised the manuscript. AC collected the samples and performed the diagnostic pathology analysis and interpretation. MS designed the experiments. MP and RH analyzed the data and designed the experiments. WA, SF, and TB performed the 16S experiments, analyzed the data, and wrote the manuscript. All authors read and approved the final manuscript.

## Conflict of Interest Statement

SF has disclosed a significant financial interest in NuGUT LLC. In accordance with its conflict of interest policy, the University of Nebraska–Lincoln’s Conflict of Interest in Research Committee has determined that this must be disclosed. MN has a patent pending related to this research. The remaining authors declare that the research was conducted in the absence of any commercial or financial relationships that could be construed as a potential conflict of interest.
