# Supplementary material for: Fecal Microbiota Transplantation Is Associated With Reduced Morbidity and Mortality in Porcine Circovirus Associated Disease
Source: Front Microbiol. 2018 Jul 23;9:1631. doi: 10.3389/fmicb.2018.01631 (PMC6064930; doi:10.3389/fmicb.2018.01631)
Supplement: Supplementary file 1 [file Data_Sheet_1.DOCX]

Supplementary Material

**Fecal Microbiota Transplantation is Associated with Reduced Morbidity and Mortality in Porcine Circovirus Associated Disease**

Megan C. Niederwerder, Laura A. Constance, Raymond R. R. Rowland, Waseem Abbas, Samodha C. Fernando, Megan L. Potter, Maureen A. Sheahan, Thomas E. Burkey, Richard A. Hesse, and Ada G. Cino-Ozuna

*** Correspondence:** Corresponding author: Megan C. Niederwerder, Email: [mniederwerder@vet.k-state.edu](mailto:mniederwerder@vet.k-state.edu)

Supplementary Data

# Supplementary Figures and Tables

## Supplementary Figures

**Supplementary Figure 1. 16S rDNA fecal microbiome analysis pre and post fecal microbiota transplantation.** A. Chao1 alpha diversity of the control and transplanted groups pre and post-transplantation (data is shown as the range of values with medians, quartiles and outliers). B. Bar graphs show the mean relative abundance of bacterial phyla for each group and time. C. Bar graphs show mean relative abundance of bacterial families making up 1% or more of all sequences detected in 1 or more sample subset.

**Supplementary Figure 2. Differentially abundant operational taxonomic units (OTU) in the control and FMT groups after 7 days of fecal microbiota transplantation.** In the FMT group, 73.3% of the differential OTUs belong to the *Veillonellaceae, Lachnospiraceae, and Ruminococcaceae* families, and 13.3% of the OTUs were not classified at the family level. For the control group, 40% of the differential OTUs belong to *Erysipelotrichaceae*, *Lachnospiraceae* and *Ruminococcaceae* families and 33.3% of the differential OTUs were unclassified. Interestingly, the hierarchical clustering of the differential OTUs show two major clusters for each of the control and FMT groups.

**Supplementary Figure 3. Fecal bacterial diversity in the PCVAD-affected and unaffected pigs at the time of challenge.** Data is shown as A. Chao1 alpha diversity and B. Observed OTUs for the affected and unaffected pigs after transplantation or mock-transplantation (data is shown as the range of values with medians, quartiles and outliers). PCVAD-affected pigs developed disease and were euthanized or died due to severity of clinical signs during the 42-day post-infection trial.
