# Supplementary figures and images for: Fecal Microbiota Transplantation Is Associated With Reduced Morbidity and Mortality in Porcine Circovirus Associated Disease
Source: Front Microbiol. 2018 Jul 23;9:1631. doi: 10.3389/fmicb.2018.01631 (PMC6064930; doi:10.3389/fmicb.2018.01631)

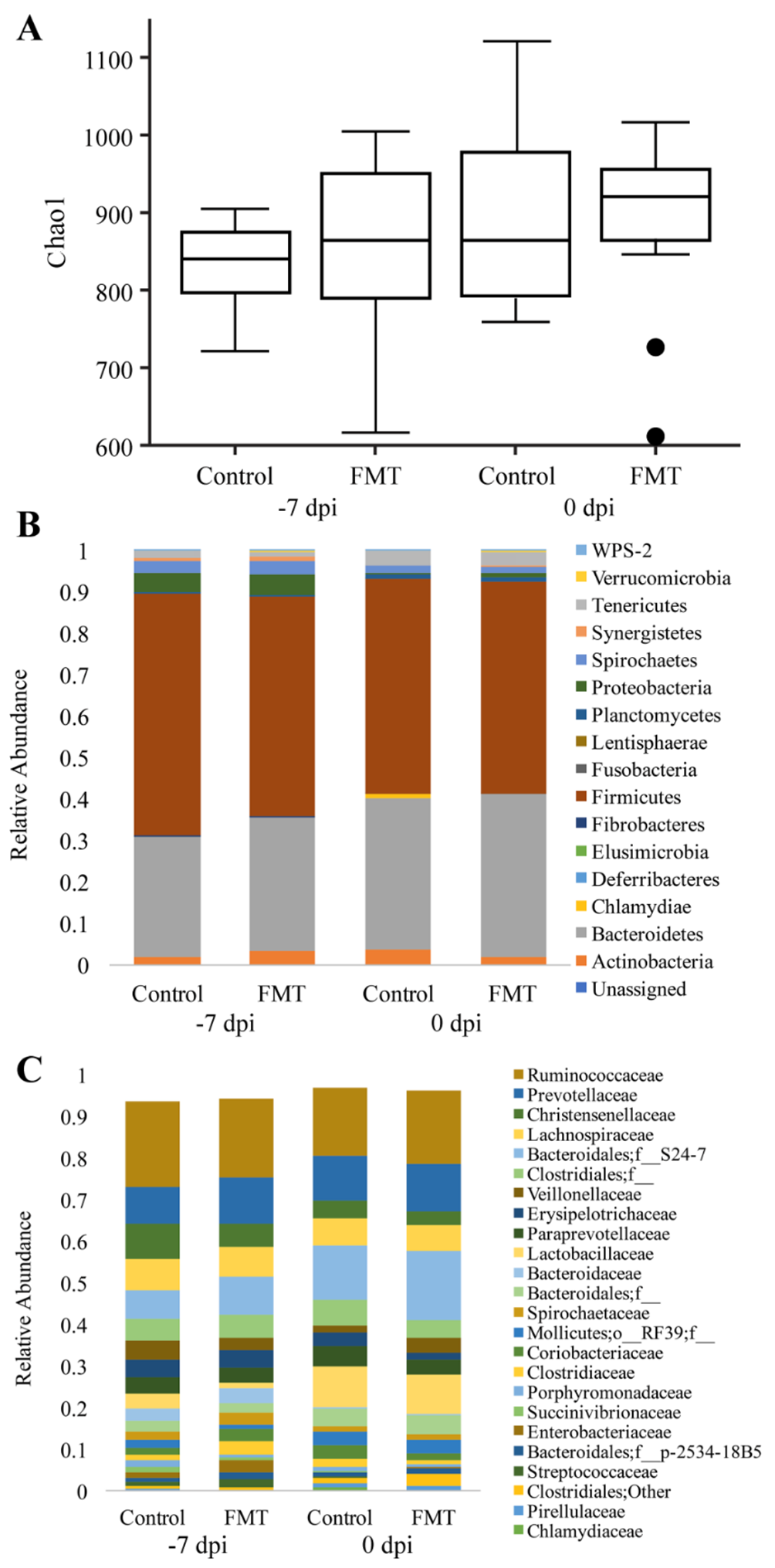

Supplement: Supplementary file 2 [file Image_1.TIF]

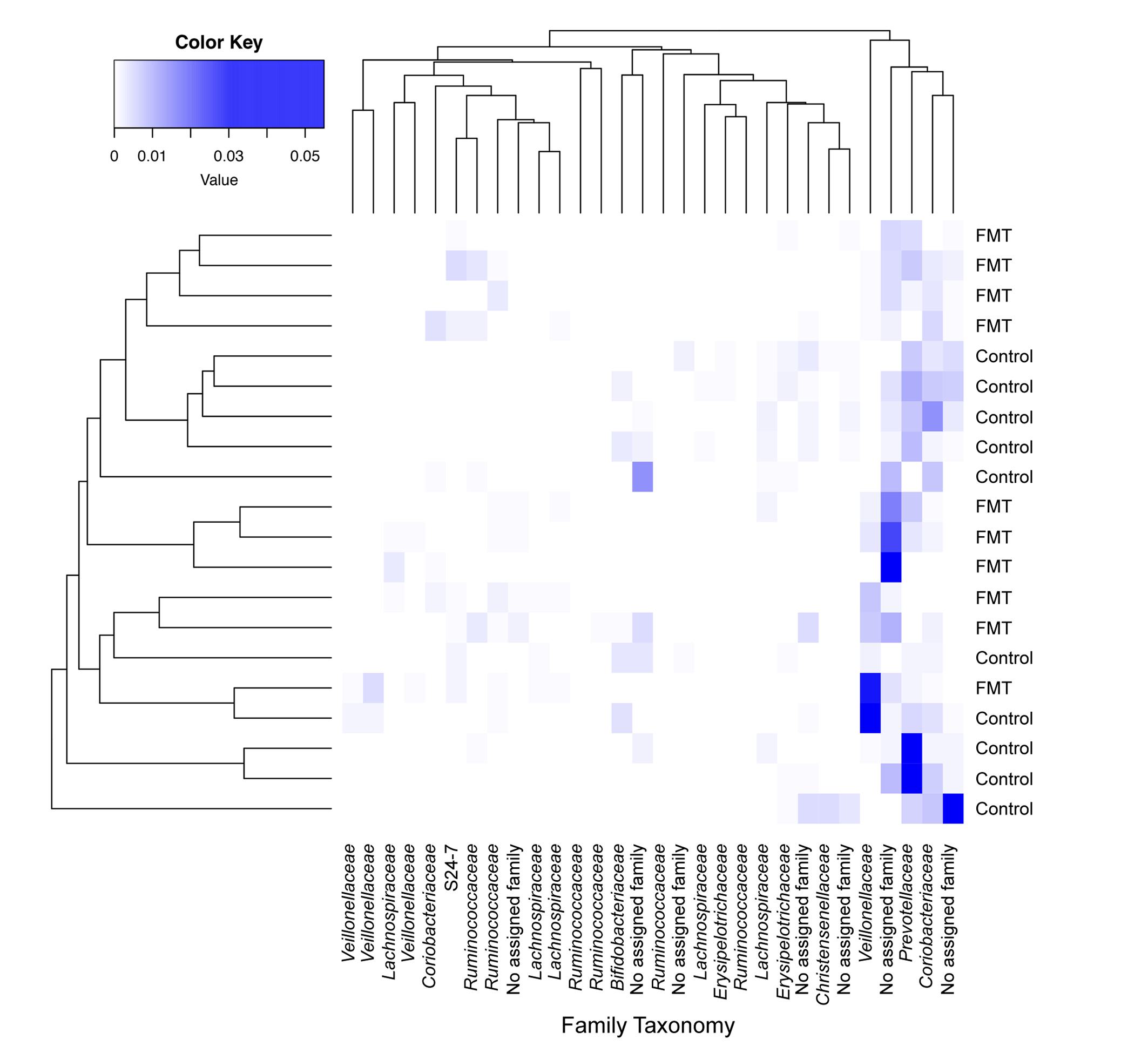

Supplement: Supplementary file 3 [file Image_2.TIF]

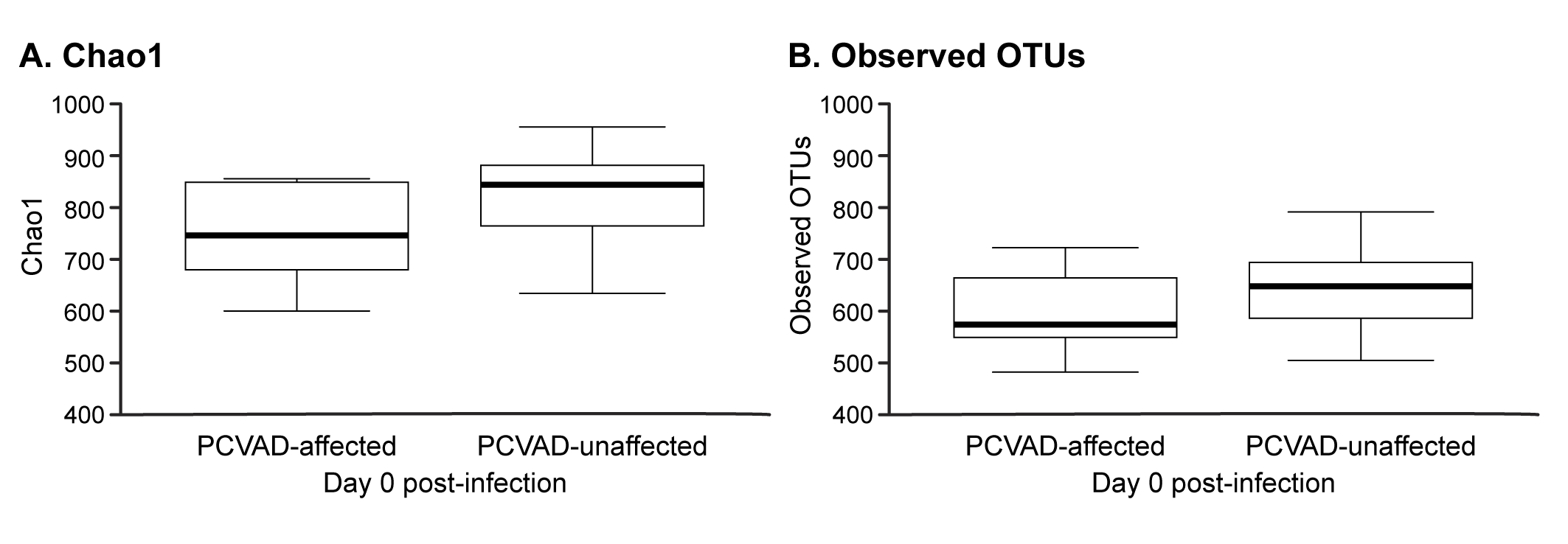

Supplement: Supplementary file 4 [file Image_3.TIF]
